# An area under the concentration–time curve threshold as a predictor of efficacy and nephrotoxicity for individualizing polymyxin B dosing in patients with carbapenem-resistant gram-negative bacteria

**DOI:** 10.1186/s13054-022-04195-7

**Published:** 2022-10-18

**Authors:** Jing Yang, Shaohua Liu, Jingli Lu, Tongwen Sun, Peile Wang, Xiaojian Zhang

**Affiliations:** 1 Department of Pharmacy, First Affiliated Hospital of Zhengzhou University, No. 1 Jianshe East Road, Zhengzhou, Henan 45005 People’s Republic of China; 2 Henan Key Laboratory of Precision Clinical Pharmacy, Zhengzhou University, Zhengzhou, People’s Republic of China; 3 Henan Engineering Research Center for Application and Translation of Precision Clinical Pharmacy, Zhengzhou University, Zhengzhou, People’s Republic of China; 4 Department of General Intensive Care Unit, First Affiliated Hospital of Zhengzhou University, Zhengzhou, People’s Republic of China

**Keywords:** Polymyxin B, AUC_ss,24 h_ threshold, Nephrotoxicity, Efficacy, Therapeutic drug monitoring

## Abstract

**Background:**

Evidence supports therapeutic drug monitoring of polymyxin B, but clinical data for establishing an area under the concentration–time curve across 24 h at steady state (AUC_ss,24 h_) threshold are still limited. This study aimed to examine exposure–response/toxicity relationship for polymyxin B to establish an AUC_ss,24 h_ threshold in a real-world cohort of patients.

**Methods:**

Using a validated Bayesian approach to estimate AUC_ss,24 h_ from two samples, AUC_ss,24 h_ threshold that impacted the risk of polymyxin B-related nephrotoxicity and clinical response were derived by classification and regression tree (CART) analysis and validated by Cox regression analysis and logical regression analysis.

**Results:**

A total of 393 patients were included; acute kidney injury (AKI) was 29.0%, clinical response was 63.4%, and 30-day all-cause mortality was 35.4%. AUC_ss,24 h_ thresholds for AKI of > 99.4 mg h/L and clinical response of > 45.7 mg h/L were derived by CART analysis. Cox and logical regression analyses showed that AUC_ss,24 h_ of > 100 mg h/L was a significant predictor of AKI (HR 16.29, 95% CI 8.16–30.25, *P* < 0.001) and AUC_ss,24 h_ of ≥ 50 mg h/L (OR 4.39, 95% CI 2.56–7.47, *P* < 0.001) was independently associated with clinical response. However, these exposures were not associated with mortality. In addition, the correlation between trough concentration (1.2–2.8 mg/L) with outcomes was similar to AUC_ss,24 h_.

**Conclusions:**

For critically ill patients, AUC_ss,24 h_ threshold of 50–100 mg h/L was associated with decreased nephrotoxicity while assuring clinical efficacy. Therapeutic drug monitoring is recommended for individualizing polymyxin B dosing.

**Supplementary Information:**

The online version contains supplementary material available at 10.1186/s13054-022-04195-7.

## Background

Polymyxins (i.e. colistin and polymyxin B) are old antibiotics that have been reintroduced in clinical practice because of the increasing incidence rate of carbapenem-resistant gram-negative bacteria (CR-GNB) infections [1, 2]. However, nephrotoxicity is the most common adverse effect of polymyxins, with rates of acute kidney injury (AKI) ranging widely from 20 to 60% [3].

Pharmacokinetics (PK)/pharmacodynamics (PD) theory can effectively promote the rational use of antibiotics [4]. As an agent with wide inter-individual variability in PK and a narrow therapeutic index, polymyxin B is an ideal candidate for therapeutic drug monitoring (TDM) [5, 6]. An area under the concentration–time curve across 24 h at steady state (AUC_ss,24 h_) of 50–100 mg h/L is recommended for the treatment of CR-GNB with minimum inhibitory concentration (MIC) values of ≤ 2 mg/L, focusing on *Acinetobacter baumannii*, *Pseudomonas aeruginosa*, and Enterobacteriaceae [7, 8]. However, it should be pointed out that the upper and lower bound of this therapeutic window was derived from a pharmacometrics meta-analysis of polymyxin B nephrotoxicity data and murine thigh infection PK/PD studies [9, 10]. Therefore, it is necessary to re-evaluate whether this threshold applies to critically ill patients.

Several studies have analyzed predictors for nephrotoxicity/efficacy and population PK of polymyxin B and reported dose as an independent predictor of AKI. Once daily dose is associated with AKI, it is expected that polymyxin B concentrations also be associated with AKI. However, a direct correlation between polymyxin B exposure and response/toxicity has not been well demonstrated [11–15]. Previously, we found that AUC_ss,24 h_ of > 100 mg h/L was a good predictor of the probability of nephrotoxicity (*P* = 0.001) [16]. Ye et al. found that the therapeutic target of AUC_ss,24 h_ (odds ratio [OR] = 13.15, *P* = 0.015) was independently associated with favorable clinical outcomes of polymyxin B treatment [17]. Some studies reported that maintaining a trough concentration (C_0h_ or C_min_) of polymyxin B below 3.13 mg/L or peak concentration (C_max_) of polymyxin B1 below 5.23 mg/L might help reduce the incidence of polymyxin B-related nephrotoxicity [18, 19]. After all, C_0h_ is usually an appropriate choice for antibiotics with AUC/MIC as PK/PD index, such as linezolid, amikacin, and voriconazole [20]. However, considering the small sample size, further research is needed to validate the exposure target extensively.

The primary objective of this study was to validate an AUC_ss,24h_ threshold of polymyxin B for predicting nephrotoxicity and efficacy in patients with CR-GNB infections. We also compared the effectiveness of AUC_ss,24h_ and C_0h_. In addition, risk factors for AKI, clinical efficacy, and mortality were evaluated.

## Methods

### Patient enrollment and data collection

This retrospective observational study was performed at the first affiliated hospital of Zhengzhou University. From April 2018 to March 2022, all patients (≥ 18 years) were included if they had received intravenous polymyxin B (sulfate; polymyxin B injection, Shanghai First Biochemical Pharmaceutical Co., Ltd., China) for the treatment of CR-GNB infections and had TDM. The susceptibility to carbapenems of the causative GNB was determined according to the European Committee on Antimicrobial Susceptibility Testing (EUCAST). Enterobacteriaceae with MIC ≥ 4 mg/L and *P. aeruginosa* and *Acinetobacter* spp. with MIC ≥ 8 mg/L were considered resistant to carbapenem [21]. The EUCAST breakpoints of polymyxin B for the above bacterial strains were 2 mg/L or lower for susceptible and > 2 mg/L for resistant [8]. Polymyxin-sensitive bacteria were performed using a VITEK® 2 COMPACT automated system (bioMérieux, Marcy-l’Étoile, France) with VITEK cards (0.5–16 mg/L for polymyxin). Exclusion criteria were (i) less than 18 years of age; (ii) received polymyxin B administration less than 96 h; (iii) received renal replacement therapy prior to polymyxin B therapy; iv) had no pathogenic microorganism result; and (v) had wrong blood drawing time. This study was approved by the Ethics Committees of the First Affiliated Hospital of Zhengzhou University (2020-KY-0318) and waived informed consent given the retrospective nature.

Data were extracted from electronic medical records, including demographics, comorbidities, medication therapy, physiological parameters, and laboratory values on the day of polymyxin B initiation. In addition, severity of illness was quantified using the Acute Physiology and Chronic Health Enquiry (APACHE II) score and the Sequential Organ Failure Assessment (SOFA) score using the worst physiological parameters within 24 h before polymyxin B initiation [22, 23]. Diagnoses of infection site were based on clinical features and positive culture of CR-GNB in sterile localized, and the absence of any bacterial pollution or colonization by two physicians.

### Polymyxin B administration and concentration determination

Polymyxin B package insert recommended doses of 1.5–3.0 mg/kg/day in two divided administrations. Therapy management was at the discretion of physicians, including dosage, infusion time, administration period, adjustment treatment, and concomitant antibiotics. Concomitant antibiotics referred to the use of other antibiotics with different mechanisms against pathogenic microorganisms to improve efficacy during polymyxin B treatment. The type and treatment course of concomitant antibiotics were based on pathogen susceptibility results, clinical features, and Chinese consensus statements [24], and only that administered for at least 72 h during polymyxin B treatment were recorded. Moreover, the following medications or medication classes were considered potential nephrotoxic drugs: vancomycin (≥ 1.0 mg/day), aminoglycoside, amphotericin B, furosemide (≥ 20 mg/day), vasoactive drugs, and immunosuppressant, which should be concomitantly used at any time during polymyxin B therapy for at least 72 h.

Polymyxin B plasma concentrations were obtained as part of our hospital's routine clinical practice of TDM. TDM was assessed first on day 4 and then repeated 48 h after dose adjustment. Dose adjustment was determined by physicians based on clinical features, MIC results, and the levels. Only the last TDM results were included in the study. For TDM, two blood samples were collected immediately before the infusion (C_0h_) and 2 h after the beginning of infusion (C_2h_). Blood samples were immediately centrifuged at 3500 ×*g* for 10 min. The supernatant was collected and stored at − 80 °C before analysis within one week.

The plasma concentrations of polymyxin B were determined using a validated ultra-performance liquid chromatography-tandem mass spectrometry previously published in our laboratory [25]. Briefly, the assay was linear over 0.2–10.0 μg/mL for polymyxin B1 and 0.05–2.5 μg/mL for polymyxin B2. The relative standard deviation (% RSD) of intra- and inter-batch assay ranged from 0 to 13.9% for quality control samples, and their corresponding accuracy (% relative error) ranged from − 11.6 to 11.1%. Since polymyxin B1 and B2 had similar structures, molecular weight (Mol.), pharmacological activities, and PK characteristics, the concentration (Conc.) of polymyxin B was derived by the equation as Conc_(polymyxin B)_ = [Conc_(polymyxin B1)_/Mol_(polymyxin B1)_ + Conc _(polymyxin B2)_/Mol_(polymyxin B2)_] × Mol_avg(polymyxin B)_.

For each patient, AUC_ss,24 h_ was estimated using the Bayesian priors from our previously published population PK model using Phoenix® NLME software (v8.3, Pharsight, Mountain View, CA, USA) [26]. In short, the mean parameter vector and the variance–covariance matrix from a previously published two-compartment population PK model were used as the Bayesian prior, then to estimate the Bayesian conditional posterior PK parameters for each patient using the dosing, concentrations, and creatinine clearance (CrCL) values. Based on Bayesian conditional posterior PK parameters, the AUC_ss,24 h_ were estimated. This approach had been validated to assess AUC values with high precision and low bias using C_0h_ and C_2h_ only [26]. CrCL was estimated according to the Cockcroft–Gault formula [27].

### Endpoints

The primary endpoint was the occurrence of AKI, which was defined as a serum creatinine increase of 0.3 mg/L (26.5 μmol/L) and 50% from baseline on two consecutive measurements during polymyxin B treatment. Further classification was based on the Kidney Disease Improving Global Outcomes (KDIGO) criteria [28].

Secondary endpoints were clinical efficiency and 30-day all-cause mortality. Clinical response was considered at the end of treatment by two physicians: disappearance or improvement of clinical symptoms (body temperature < 38.0 °C), radiological resolution of signs of infection, and improved biochemistry indicators of infection (a ≥ 30% decrease in the total peripheral white blood cells count or C-reactive protein level). Patients who did not meet all above criteria were classified as clinical failure. Thirty-day mortality was recorded from the start of polymyxin B treatment.

### Statistical analysis

Statistical analyses were performed using the Statistical Package for the Social Sciences version 26.0 (SPSS Inc., Chicago, IL, USA). Data were expressed as median with interquartile range (IQR) for continuous variables and percentages/frequency (%) for categorical variables. Categorical variables were analyzed by the chi-square test. Normally distributed variables were analyzed by the Student’s *t* test, while non-normally distributed variables were analyzed by Mann–Whitney test. Variables (Table 1 and infection sites) with *P* values of < 0.1 were included in Cox proportional hazards model to estimate hazard ratios (HRs) for AKI and mortality, and logistic regression model to estimate odds ratios (ORs) for clinical response. The effect of collinearity among variables (tolerance < 0.2 or variance inflation factor > 10) was eliminated in the models. Classification and regression tree (CART) method was used to split the samples into subgroups based on chi-square statistics. Thirty-day mortality and polymyxin B-associated AKI were compared with Kaplan–Meier analysis and log-rank test. *P* value of < 0.05 was considered significant.

**Table 1:** Patient characteristics

Variable	All (*n* = 393)
Age, years	56.0 (48.0–65.0)
Male, n (%)	287 (73.0%)
Weight, kg	70.0 (60.0–75.0)
BMI, kg/m^2^	23.7 (21.2–25.7)
ICU admission, n (%)	361 (91.9%)
Mechanical ventilation, n (%)	269 (68.4%)
SOFA score	8.0 (6.0–10.0)
APACHE II score	18.0 (13.0–23.0)
*Comorbidities, n (%)*	
Diabetes	127 (32.3%)
Malignancy	53 (13.5%)
Hypertension	162 (41.2%)
Heart disease	97 (24.7%)
Stroke	92 (23.4%)
Sepsis	186 (47.3%)
Septic shock	153 (38.9%)
*Pathogen, n (%)*	
*Klebsiella pneumoniae*	177 (42.7%)
*Acinetobacter baumannii*	168 (40.5%)
*Pseudomonas aeruginosa*	44 (10.6%)
*Escherichia coli*	17 (4.1%)
*Pneumogenic klebsiella*	9 (2.2%)
*Polymyxin B treatment*	
Duration days	13.0 (8.0–18.0)
Daily dose, mg	150.0 (100.0–150.0)
Daily dose/weight, mg/kg/day	2.12 (1.67–2.50)
*Concomitant antibiotics, n (%)*	
Carbapenem	209 (53.2%)
Cephalosporin	184 (46.8%)
Tigecycline	136 (34.6%)
Rifampicin	25 (6.4%)
Aminoglycosides	12 (3.1%)
Fosfomycin	8 (2.0%)
Aztreonam	7 (1.8%)
*Laboratory data*	
GFR, mL/min·1.73m^2^	101.2 (71.3–118.3)
Scr, µmol/L	62.0 (45.3–93.4)
Albumin, g/L	30.0 (27.2–34.9)
White blood cell, 10^9^/L	11.2 (8.0–15.7)
Platelets, 10^9^/L	168.5 (88.0–275.3)
C-reactive protein, μg/L	72.2 (34.6–140.0)
Procalcitonin, ng/mL	1.0 (0.4–3.6)
*Concomitant nephrotoxic drugs, n (%)*	
Vancomycin	83 (21.1%)
Aminoglycoside	12 (3.1%)
Amphotericin B	35 (8.9%)
Furosemide	154 (39.2%)
Vasoactive drugs	195 (50.5%)
Immunosuppressant	32 (8.1%)
Use of nephrotoxic drugs^a^	289 (73.5%)
AUC_ss,24 h_, mg h/L	58.5 (40.6–77.2)
C_0h_, mg L^−1^	1.24 (0.74–1.93)

Data were n (%) or median (interquartile range, IQR)

*AKI* acute kidney injury; *BMI* body mass index; *ICU* intensive care unit; *SOFA* Sequential Organ Failure Assessment; *APACHE II* Acute Physiology, Age, Chronic Health Evaluation II; *GFR* glomerular filtration rate; *Scr* serum creatinine; *AUC*_*ss*,24* h*_, the area under the curve across 24 h at steady state; *and C*_*0h*_ concentration pre-dose

^a^nephrotoxic drug including vancomycin, aminoglycoside, amphotericin B, furosemide, vasoactive drugs, and immunosuppressant

## Results

### Patients Characteristics

**Fig. 1:**
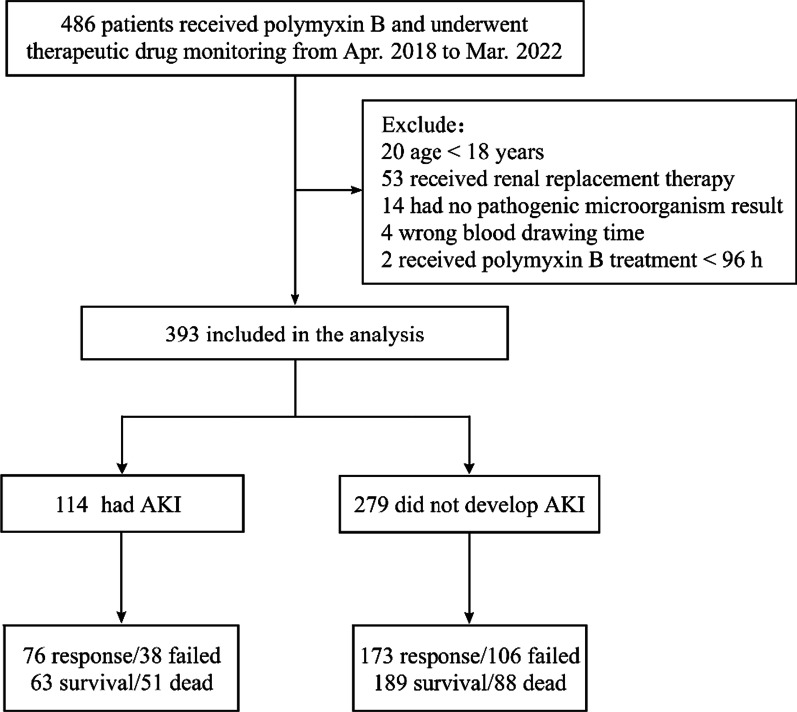
Flow diagram of patient inclusion and exclusion. AKI, acute kidney injury

A total of 486 patients received polymyxin B and had TDM during the study period. Ninety-three of them were excluded, and 393 patients were eventually included in the follow-up analysis (Fig. 1). The median age of the patients was 56 years old (IQR, 48–65 years), and 73.0% were male. Among them, 68 patients (17.3%) had multi-site infections. The highest infection site was lung (305/393), followed by bloodstream (96/393), abdomen (26/393), cerebral (17/393), urinary tract (11/393), and skin and soft tissue (6/393). Polymyxin-sensitive bacteria were observed in all patients with MICs ≤ 0.5 mg/L. Information on patient demographics, indications for therapy, and underlying conditions is presented in Table 1.

### Polymyxin B exposure and trough concentrations

**Fig. 2:**
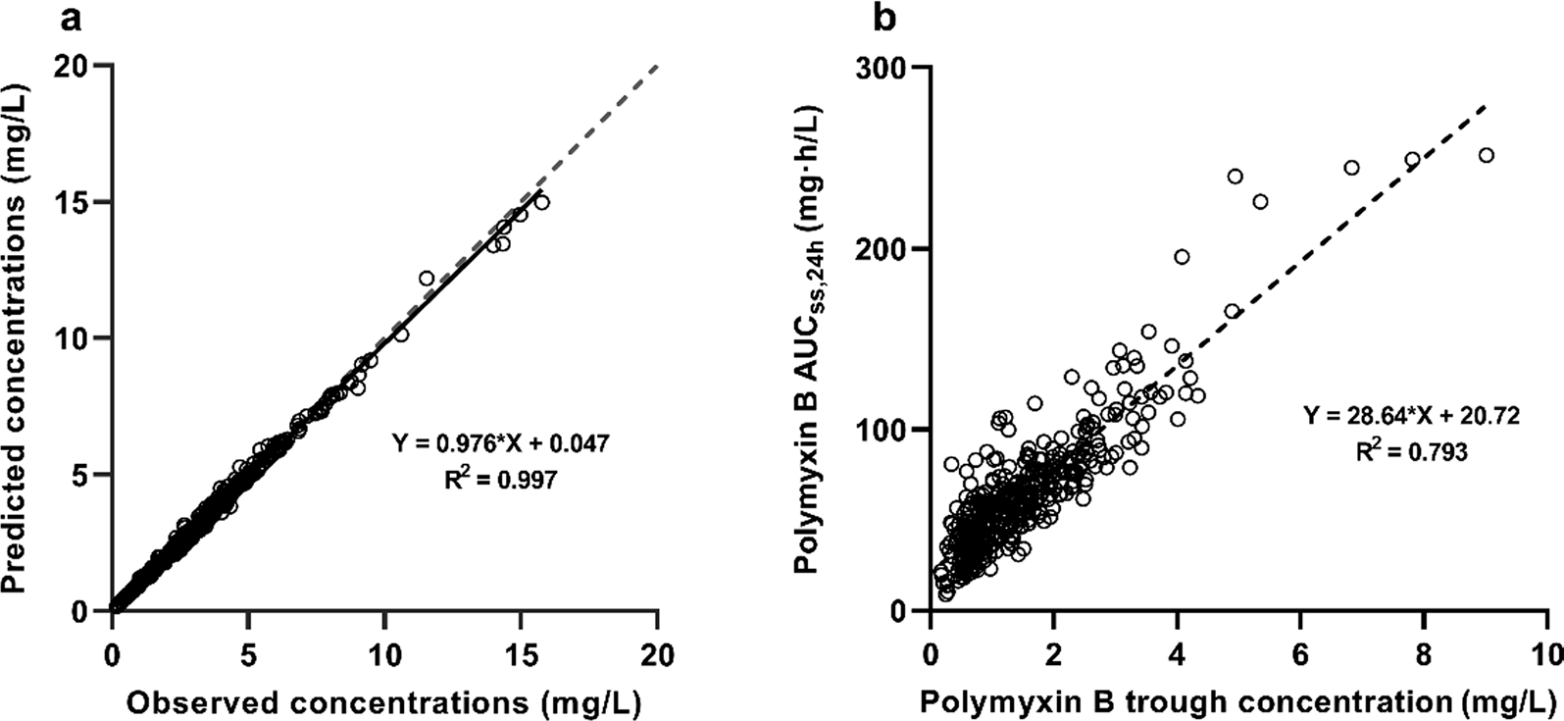
Scatterplot of polymyxin B concentrations. Observed versus predicted polymyxin B concentration for Bayesian estimation approach (**a**). Bayesian estimated area under the curve across 24 h at steady state (AUC_ss,24 h_) versus trough concentration (**b**)

Observed and predicted polymyxin B concentrations from Bayesian estimation are shown in Fig. 2a. The coefficient of determination (R^2^) of regression line was 0.997, which indicated the predictive performance of Bayesian approach. The median maintenance dose was 2.12 mg/kg/day (IQR, 1.67–2.50 mg/kg/day), which resulted in a median AUC_ss,24 h_ of 58.5 mg h/L (IQR, 40.6–77.2 mg h/L) and a median C_0h_ of 1.24 mg/L (IQR, 0.74–1.93 mg/L). Figure 2b shows a positive correlation between C_0h_ and AUC_ss,24 h_ with an R^2^ of 0.793 was detected. Additionally, polymyxin B exposure increased with daily dosage, but it varied largely from patient to patient (Additional file 1: Figure S1).

### AKI

**Fig. 3:**
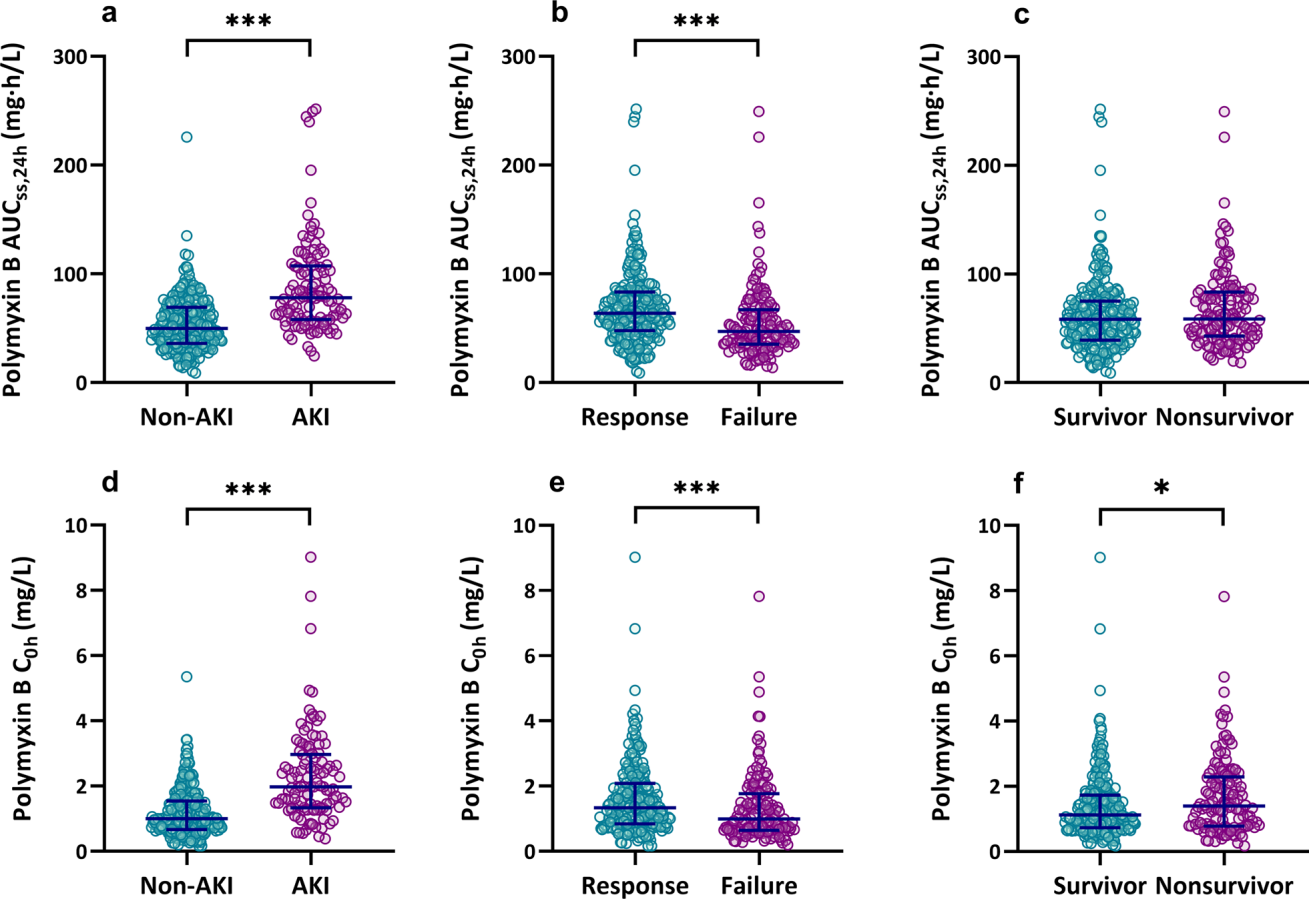
Scatterplot of polymyxin B AUC_ss,24 h_ and C_0h_ stratified for outcomes. AUC_ss,24 h_ for AKI (**a**), clinical efficacy (**b**), and survival (**c**); C_0h_ for AKI (**d**), clinical efficacy (**e**), and survival (**f**). AUC_ss,24 h_, the area under the plasma concentration–time curve across 24 h at steady state; C_0h_, trough concentration; AKI, acute kidney injury; ***represent *P* < 0.001; and *represent *P* < 0.05

AKI was observed in 29.0% of patients (114/393). Of these, 51 (44.7%) were classified as stage 1; 34 (29.8%) as stage 2; and 29 (25.4%) as stage 3. The median time to develop AKI was 8 days (IQR, 5–14 days). The median AUC_ss,24 h_ was significantly higher (78.2 mg h/L; IQR, 58.1–107.5 mg h/L) in patients who developed AKI than in those did not (50.6 mg h/L; IQR, 36.1–69.5 mg h/L; *P* < 0.001, Fig. 3a). As for AKI degrees (Additional file 1: Figure S2), AUC_ss,24 h_ of stage 3 was slightly higher than that of stage 1 (*P* = 0.044).

**Fig. 4:**
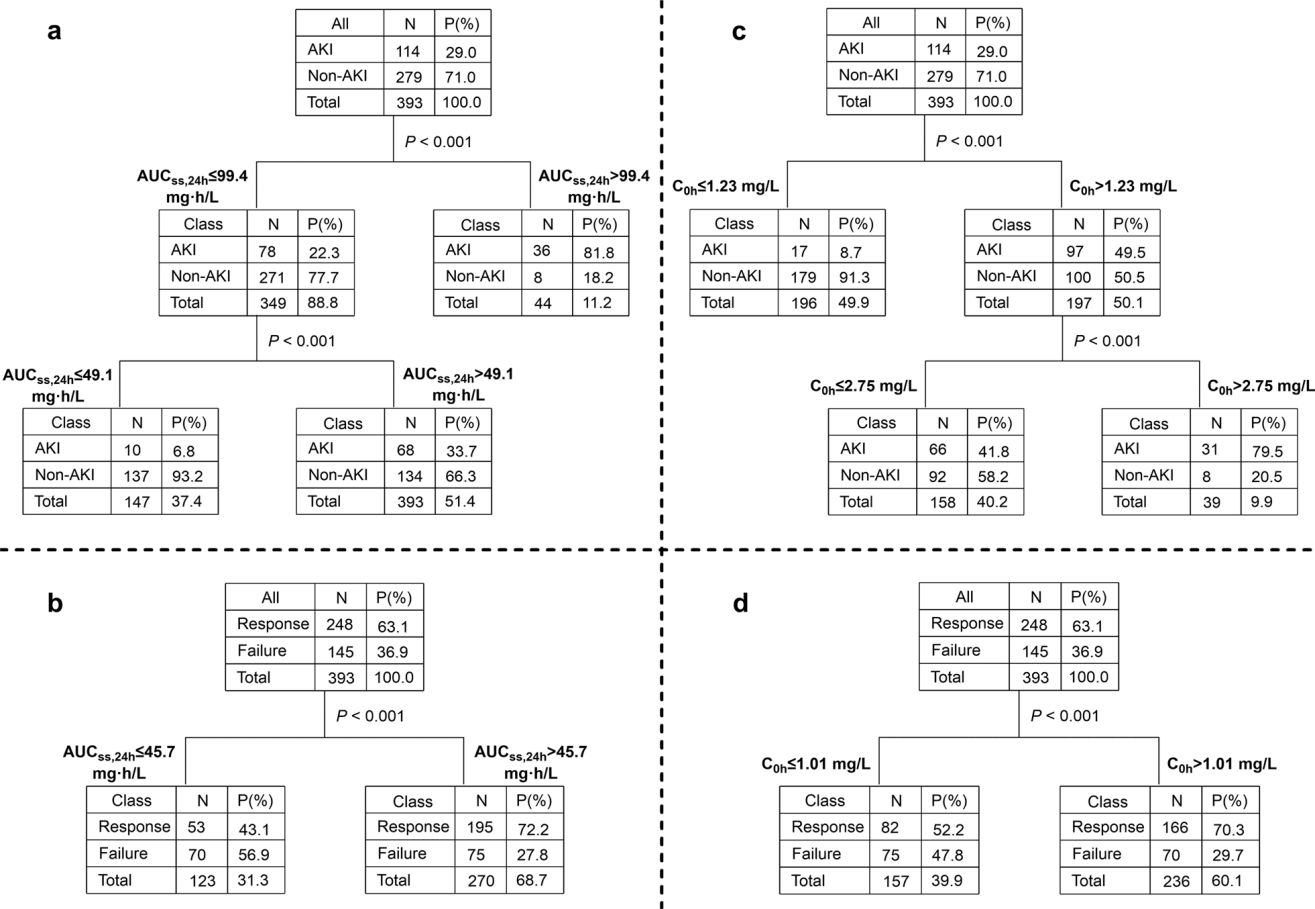
Classification and regression tree results for the incidence of AKI and clinical efficacy. AUC_ss,24 h_ for AKI (**a**) and clinical efficacy (**b**); C_0h_ for AKI (**c**) and clinical efficacy (**d**). AKI, acute kidney injury; AUC_ss,24 h_, the area under the plasma concentration–time curve across 24 h at steady state; and C_0h_, trough concentration

CART analysis (Fig. 4a) revealed that AUC_ss,24 h_ of > 99.4 mg h/L was significantly associated with AKI (*P* < 0.001). Then, subgroup analysis showed that patients with AUC_ss,24 h_ of > 49.1 mg h/L had a higher risk of AKI than those with AUC_ss,24 h_ of ≤ 49.1 mg h/L (*P* < 0.001). These results were consistent with the target AUC_ss,24 h_ window of 50–100 mg h/L [7], and accordingly, the following analysis took concentrations of 50 mg h/L and 100 mg h/L as cutoff points.

**Fig. 5:**
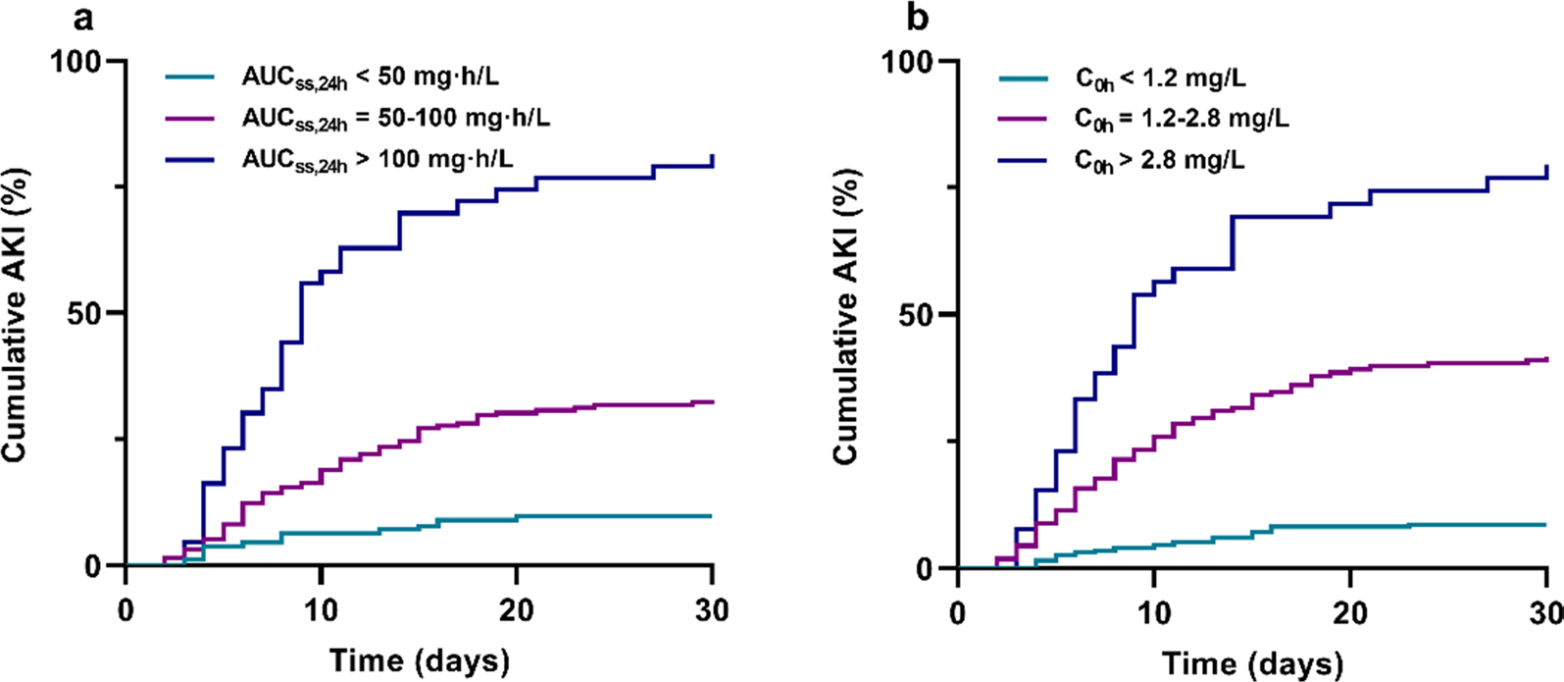
Kaplan–Meier estimates of the incidence of AKI to day 30 after administration of polymyxin B. Stratified by AUC_ss,24 h_ (**a**) and C_0h_ (**b**). AKI, acute kidney injury; AUC_ss,24 h_, the area under the plasma concentration–time curve across 24 h at steady state; and C_0h_, trough concentration

In both of Kaplan–Meier analysis (log-rank, *P* < 0.001, Fig. 5a) and Cox regression model (Table 2), AKI rate in AUC_ss,24 h_ of > 100 mg h/L group (81.4%; HR 16.29, 95% CI 8.16–30.25, *P* < 0.001) was remarkably higher than that in AUC_ss,24 h_ of 50–100 mg h/L group (32.8%; HR 3.89, 95% CI 2.17–6.97, *P* < 0.001) and AUC_ss,24 h_ of < 50 mg h/L group (9.7%, *P* < 0.001).

**Table 2:** Univariate and Cox regression model for AKI

Variable	No AKI (*n* = 279)	AKI (*n* = 114)	*P*^a^	HR (95% CI)	*P*^b^
Age, years	54.0 (47.0–64.0)	58.0 (50.8–66.0)	0.012	1.02 (1.01–1.03)	0.003
GFR, mL/min·1.73 m^2^	103.1 (74.0–121.3)	95.5 (66.2–110.6)	0.025		
Furosemide	101 (36.2%)	53 (46.5%)	0.058		
Use of nephrotoxic drugs	196 (70.3%)	93 (81.6%)	0.021		
*Polymyxin B dose and exposure*					
Dose/weight, mg/kg/day	2.0 (1.67–2.37)	2.31 (1.94–2.61)	< 0.001		
Daily dose ≥ 150 mg/day	178 (63.8%)	89 (78.1%)	0.006		
^c^AUC_ss,24 h_ < 50 mg h/L	140 (50.2%)	15 (13.2%)	< 0.001	–	< 0.001
AUC_ss,24 h_ = 50–100 mg h/L	131 (47.0%)	64 (56.1%)		3.89 (2.17–6.97)	< 0.001
AUC_ss,24 h_ > 100 mg h/L	8 (2.9%)	35 (30.7%)		16.29 (8.16–30.25)	< 0.001
^d^C_0h_ < 1.2 mg/L	173 (62.0%)	17 (14.9%)	< 0.001		
C_0h_ = 1.2–2.8 mg/L	98 (35.1%)	66 (57.9%)			
C_0h_ > 2.8 mg/L	8 (2.9%)	31 (27.6%)			

*AKI* acute kidney injury; *HR* hazard ratio; *CI* confidence interval; *GFR* glomerular filtration rate; and *AUC*_*ss,24 h*_ the area under the curve across 24 h at steady state

^a^derived from univariate analysis

^b^derived from Cox regression analysis

^c^AUC_ss,24 h_ < 50 mg h/L was taken as reference

^d^C_0h_ was not included in the Cox regression model due to collinearity with AUC_ss,24 h_

In the case of C_0h_, the median C_0h_ was significantly higher (1.98 mg/L; IQR, 1.34–2.97 mg/L) in patients who developed AKI than in those did not (1.01 mg/L; IQR, 0.67–1.54 mg/L; *P* < 0.001, Fig. 3d). CART analysis showed that two cutoff values of 1.23 mg/L and 2.75 mg/L split C_0h_ levels into three nodes based on AKI rate (Fig. 4c). For the sake of convenient use in the clinic, the values were rounded to 1.2 mg/L and 2.8 mg/L in Kaplan–Meier analysis (log-rank, *P* < 0.001, Fig. 5b).

### Clinical efficacy

The overall clinical response rate was 63.4% (249/393). The median duration of polymyxin B treatment in patients who achieved clinical response or did not was 11 and 14 days, respectively. A higher AUC_ss,24 h_ (63.9 mg h/L; IQR, 47.8–84.0 mg h/L) was observed in patients with clinical response than in those with clinical failure (47.0 mg h/L; IQR, 34.3–62.8 mg h/L; *P* < 0.001, Fig. 3b). CART analysis (Fig. 4b) revealed that a cutoff value of 45.7 mg h/L split AUC_ss,24 h_ levels into two nodes based on clinical response rate (*P* < 0.001), which was also in agreement with the lower limit of the target therapeutic window (50 mg h/L) [7, 9]. Logistic regression analysis (Table 3) showed that AUC_ss,24 h_ ≥ 50 mg h/L (OR 4.39, 95% CI 2.56–7.47, *P* < 0.001) was an independent factor associated with clinical response, as were concomitant nephrotoxic drugs, age, and septic shock.

**Table 3:** Univariate and logistic regression model for clinical response

Variable	Failure (*n* = 144)	Response (*n* = 249)	*P*^a^	OR (95% CI)	*P*^b^
Age, years	58.0 (51.0–67.0)	54.0 (47.0–63.0)	0.009	0.98 (0.96–1.0)	0.012
SOFA score	8.0 (7.0–12.0)	8.0 (5.0–10.0)	0.003		
APACHE II score	19.0 (14.0–23.0)	17.0 (11.0–21.0)	0.001		
Septic shock	69 (47.6%)	84 (33.9%)	0.007	0.55 (0.33–0.93)	0.025
Vasoactive drugs	87 (60.8%)	108 (44.4%)	0.002		
Use of nephrotoxic drugs	117 (80.7%)	172 (69.4%)	0.014	0.44 (0.24–0.83)	0.011
*Laboratory data*					
GFR, mL/min·1.73m^2^	96.0 (51.8–114.4)	102.7 (73.7–118.0)	0.049		
Albumin, g/L	29.2 (26.2–33.2)	30.5 (27.3–35.0)	0.080		
Platelets, 10^9^/L	139.0(73.0–244.0)	172.5 (85.5–271.0)	0.008		
C-reactive protein, μg/L	89.4 (45.9–150.0)	67.6 (30.8–130.7)	0.072		
Procalcitonin, ng/mL	1.2 (0.5–4.0)	0.9 (0.3–3.5)	0.080		
*Polymyxin B dose and exposure*					
Dose/weight, mg/kg/day	2.0 (1.6–2.3)	2.1 (1.8–2.5)	0.011		
Daily dose ≥ 150 mg/day	82 (56.6%)	185 (74.6%)	< 0.001		
AUC_ss,24 h_ ≥ 50 mg h/L	63 (43.4%)	175 (70.6%)	< 0.001	4.39 (2.56–7.47)	< 0.001
^c^C_0h_ > 1.01 mg/L	79 (54.9%)	169 (67.9%)	< 0.001		

*OR* odds ratio; *CI* confidence interval; *GFR* glomerular filtration rate; and *AUC*_*ss,24 h*_ the area under the curve across 24 h at steady state

^a^derived from univariate analysis

^b^derived from logistic regression analysis

^c^C_0h_ was not included in the logistic regression model due to collinearity with AUC_ss,24 h_

In addition, the median C_0h_ was higher (1.35 mg/L; IQR, 0.85–2.09 mg/L) in patients with clinical response than with clinical failure (0.99 mg/L; IQR, 0.65–1.76 mg/L; *P* < 0.001, Fig. 3e), and maintenance of C_0h_ > 1.01 mg/L was identified to be correlated with clinical response (Fig. 4d).

### Mortality

The 30-day all-cause mortality was 35.4% (139/393). No significant difference in AUC_ss,24 h_ between surviving and non-surviving patients was observed (58.3 mg h/L [39.5–75.2 mg h/L] vs. 58.2 mg·h/L [43.1–83.4 mg·h/L]; *P* = 0.184, Fig. 3c). And, there was no difference within each AUC_ss,24 h_ group with regard to 30-day mortality (34.2% vs. 34.9% vs. 41.9%, log-rank, *P* = 0.785, Additional file 1: Figure S3). In contrast, C_0h_ showed statistic difference between survivors and non-survivors (1.12 mg/L [0.73–1.73 mg/L] vs. 1.40 mg/L [0.78–2.29 mg/L]; *P* = 0.018, Fig. 3f). Only septic shock, glomerular filtration rate, heart disease, and use of nephrotoxic drugs were associated with 30-day mortality in Cox regression model (Table 4).

**Table 4:** Univariate and Cox regression model for 30-day mortality

Variable	Survival (*n* = 254)	No survival (*n* = 139)	*P*^a^	HR (95% CI)	*P*^b^
Age, years	54.0 (48.0–63.0)	59.0 (48.0–68.0)	< 0.001		
ICU admission, n (%)	229 (90.2%)	132 (95.0%)	0.096		
Mechanical ventilation	163 (64.2%)	106 (76.3%)	0.014		
SOFA score	8.0 (5.0–10.0)	9.0 (7.0–12.0)	< 0.001		
APACHE II score	17.0 (11.0–21.8)	19.0 (14.0–24.0)	0.002		
*Comorbidities*					
Diabetes	74 (29.1%)	53 (38.1%)	0.068		
Heart disease	46 (18.1%)	51 (36.7%)	< 0.001	1.92 (1.31–2.83)	0.001
Sepsis	106 (41.7%)	80 (57.6%)	0.003		
Septic shock	75 (29.5%)	78 (56.1%)	< 0.001	2.29 (1.54–3.40)	< 0.001
*Laboratory data*					
GFR, mL/min·1.73m^2^	103.1 (80.6–118.9)	86.5 (41.8–110.9)	< 0.001	0.99 (0.99–1.0)	0.001
Albumin, g/L	30.2 (27.3–34.9)	29.2 (26.6–32.9)	0.021		
Platelets, 10^9^/L	178.0(84.3–294.8)	127.0 (71.0–194.0)	< 0.001		
C-reactive protein, μg/L	68.1 (30.4–127.2)	107.5 (47.3–159.9)	0.001		
Procalcitonin, ng/mL	0.7 (0.3–3.4)	1.4 (0.6–5.9)	< 0.001		
*Concomitant nephrotoxic drugs*					
Vancomycin	43 (16.9%)	40 (28.8%)	0.006		
Furosemide	89 (35.0%)	65 (47.1%)	0.020		
Vasoactive drugs	110 (43.8%)	85 (63.0%)	< 0.001		
Use of nephrotoxic drugs	166 (65.4%)	123 (88.5%)	< 0.001	2.70 (1.48–4.93)	0.001
AKI	63 (24.8%)	51 (36.7%)	0.013		

*HR* hazard ratio; *CI* confidence interval; and *GFR* glomerular filtration rate

^a^derived from univariate analysis

^b^derived from Cox regression analysis

## Discussion

This study investigated and evaluated the association between an AUC_ss,24 h_ threshold and polymyxin B exposure–response/toxicity using clinical data from critically ill patients with CR-CNB infections. As a result, a significant relationship between polymyxin B AUC_ss,24 h_ and clinical efficacy/AKI was observed. CART analysis identified two cutoff points of 99.4 mg h/L and 49.1 mg h/L as significant predictors of the incidence of AKI, and AUC_ss,24 h_ of > 45.7 mg h/L was significantly associated with clinical response (Fig. 4). These results were in accordance with the therapeutic window (50–100 mg h/L) and were also validated by regression analyses and Kaplan–Meier analysis in this study.

In the previous study, we found that an AUC_ss,24 h_ of > 100 mg h/L was a good predictor for the probability of nephrotoxicity by both the receiver operating characteristic curve and logistic regression analysis [16]. Unable to estimate AUC, Han et al. reported that C_0h_ > 3.13 mg/L significantly increased the risk of AKI [18]. According to the limited sampling strategy equation (AUC_ss,24 h_ = 21.323 + 28.189 × C_0h_) [29], the C_0h_ value (3.13 mg/L) was equivalent to an AUC_ss,24 h_ of 109.6 mg h/L. These results also supported the rationality of the upper bound of AUC_ss,24 h_ (100 mg h/L).

For antibiotics with AUC/MIC as PK/PD index, AUC better predicts efficacy and toxicity but is more challenging to get in clinical practice than C_0h_ [30, 31]. Several approaches have been proposed to estimate AUC with sparse samples, such as Bayesian method, limited sampling strategy, and Sawchuk–Zaske equation [26, 29, 32]. Using Bayesian approach, we found that C_0h_ had an apparent relationship with AUC (Fig. 2b). Moreover, CART analysis identified two cutoff points of 1.23 mg/L and 2.75 mg/L as significant predictors of the incidence of AKI (Fig. 4c) and C_0h_ of > 1.01 mg/L as a predictor for clinical response (Fig. 4d). Based on the regression equation (Fig. 2b), these C_0h_ values (1.23 mg/L, 2.75 mg/L, and 1.01 mg/L) were, respectively, equivalent to AUC_ss,24 h_ of 55.1 mg h/L, 100.9 mg h/L, and 49.6 mg h/L, which were closed to the AUC target. Since the incidence of AKI was very low when C_0h_ ≤ 1.23 mg/L (8.7%), to avoid the emergence of drug-resistant bacteria caused by low drug exposure, C_0h_ therapeutic target was simplified to 1.2–2.8 mg/L. Alternatively, in the absence of methods to calculate AUC, C_0h_ would be a credible surrogate for dosing adjustment.

Besides drug exposures, older age was also associated with polymyxin B-related AKI in Cox regression model (Table 2). Meta-analyses showed the primary factor affecting nephrotoxicity was polymyxins B dose, along with age, diabetes, and use of nephrotoxins [12, 13, 33]. Most of the above risk factors were found in the univariate analysis but had no effect in Cox regression model. This disparity was attributable to the fact that polymyxin B dose was associated with AUC_ss,24 h_, which showed a more remarkable impact on nephrotoxicity. Therefore, TDM is recommended to decrease the risk of nephrotoxicity.

Compared with AKI, polymyxin B exposures had less effect on clinical response and had no effect on 30-day mortality (Fig. 3). It was also found in the vancomycin exposure–response relationship studies [34–36]. Since this was a retrospective study, these data should be interpreted cautiously. The main reason might be that AUC/MIC ratio, not AUC, was the PK/PD parameter most closely linked to clinical outcomes [4]. In this study, polymyxin-sensitive bacteria were observed in all patients with MICs ≤ 0.5 mg/L by a VITEK® 2 COMPACT automated system rather than broth microdilution (BMD) testing. Nevertheless, Zhu et al. observed that the MICs of polymyxin in several susceptible isolates tested by the VITEK 2 system were onefold to twofold dilutions lower than those of BMD [37]. This may be the reason why the guideline recommends the use of BMD assays to assess the MICs of polymyxins [7]. Additionally, critically ill patients suffered serious underlying diseases and comorbidities, mixed infection, and a combination of multiple drugs, which also affected the eventual efficacy of polymyxin B and mortality.

This study has several limitations to be considered. First, this was a single-center retrospective study of adult, non-dialysis patients. It is unknown whether the observed findings apply to other populations. Second, the AUC threshold was delineated by CART, a useful tool to identify exposure thresholds associated with an increased risk of outcomes. Although it recognized the breakpoint that maximizes the difference in outcomes in a given study sample, it should be validated with external data. Third, this population was identified over 4 years, so unmeasured changes in CR-GNB management may result in improved outcomes over time. Known changes over this period include changes in polymyxin B dosing in accordance with consensus guidelines [7] and novel co-administered agents such as ceftazidime–avibactam [38]. Last, to truly define the polymyxin B exposure–efficacy relationship, larger-scale, multicentered prospective clinical trials are needed with the AUC/MIC analysis, which is under preparation at our center.

## Conclusions

In conclusion, the present study confirmed a significant relationship between polymyxin B AUC_ss,24 h_ and clinical efficacy/AKI in a real-world cohort of patients treated with polymyxin B for CR-GNB infections. AUC_ss,24 h_ threshold of 50–100 mg h/L was a good predictor for clinical response and AKI risk, and C_0h_ (1.2–2.8 mg/L) monitoring was also a credible surrogate for individualized dosing. Accordingly, it is necessary to recommend TDM and AUC-guided polymyxin B dosing.

## Supplementary Information

**Additional file 1: Figure S1.** Scatterplot of polymyxin B AUC_ss,24h_ versus dosage. AUC_ss,24h_, the area under the plasma concentration-time curve across 24 hours at steady state. **Figure S2.** Scatterplot of polymyxin B AUC_ss,24h_ (a) and C_0h_ (b) stratified for different stages of acute kidney injury. AUC_ss,24h_, the area under the plasma concentration-time curve across 24 hours at steady state; C_0h_, trough concentration. **Figure S3.** Kaplan-Meier estimates of survival to 30 days after administration of polymyxin B. Stratified by AUC_ss,24h_ (a) and C_0h_ (b). AUC_ss,24h_, the area under the plasma concentration-time curve across 24 hours at steady state; C_0h_, trough concentration.

## Data Availability

The datasets used during the current study are available from the corresponding author on reasonable request.

## Abbreviations

CR-GNB: Carbapenem-resistant gram-negative bacteria
AKI: Acute kidney injury
PK: Pharmacokinetics
PD: Pharmacodynamics
TD: Toxicodynamic
TDM: Therapeutic drug monitoring
AUC_ss,24__ h_: Area under the concentration–time curve across 24 h at steady state
MIC: Minimum inhibitory concentration
C_0h_ or C_min_: Trough concentration
EUCAST: European Committee on Antimicrobial Susceptibility Testing
APACHE II: Acute Physiology and Chronic Health Enquiry
SOFA: Sequential Organ Failure Assessment
C_2h_: Concentration after 2 h dose
CrCL: Creatinine clearance
KDIGO: Kidney Disease Improving Global Outcomes
IQR: Interquartile range
HR: Hazard ratio
OR: Odds ratios
CART: Classification and regression tree
R^2^: Coefficient of determination
BMD: Broth microdilution
