# Supplementary material for: An area under the concentration–time curve threshold as a predictor of efficacy and nephrotoxicity for individualizing polymyxin B dosing in patients with carbapenem-resistant gram-negative bacteria
Source: Crit Care. 2022 Oct 18;26:320. doi: 10.1186/s13054-022-04195-7 (PMC9578216; doi:10.1186/s13054-022-04195-7)
Supplement: Supplementary file 1 — Additional file 1: Figure S1. Scatterplot of polymyxin B AUCss,24h versus dosage. AUCss,24h, the area under the plasma concentration-time curve across 24 hours at steady state. Figure S2. Scatterplot of polymyxin B AUCss,24h (a) and C0h (b) stratified for different stages of acute kidney injury. AUCss,24h, the area under the plasma concentration-time curve across 24 hours at steady state; C0h, trough concentration. Figure S3. Kaplan-Meier estimates of survival to 30 days after administration of polymyxin B. Stratified by AUCss,24h (a) and C0h (b). AUCss,24h, the area under the plasma concentration-time curve across 24 hours at steady state; C0h, trough concentration. [file 13054_2022_4195_MOESM1_ESM.docx]

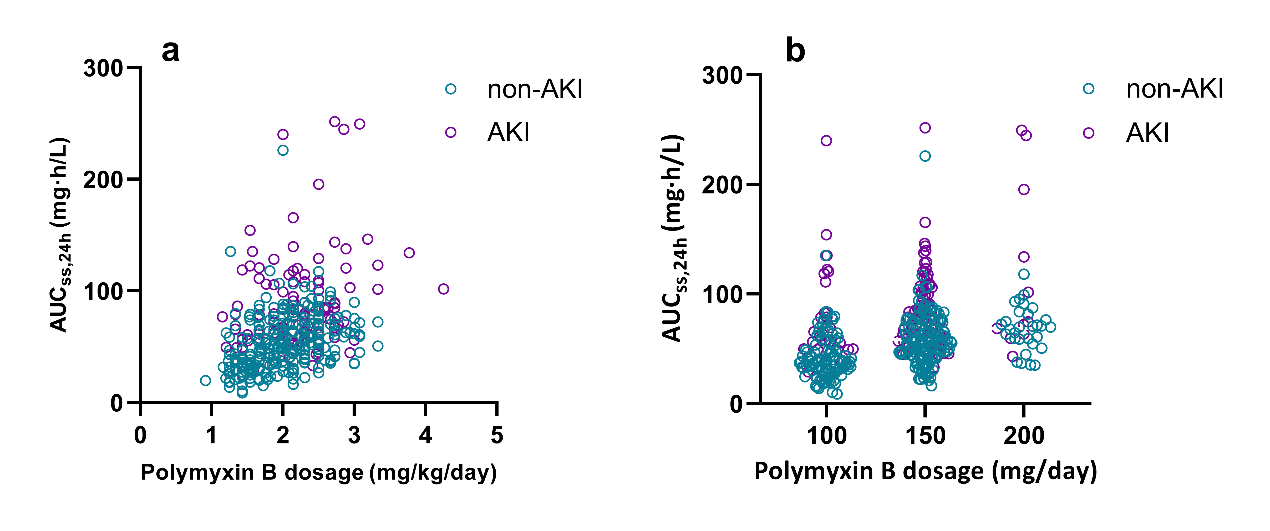


Figure S1. Scatterplot of polymyxin B AUC_ss,24h_ versus dosage. AUC_ss,24h_, the area under the plasma concentration-time curve across 24 hours at steady state.


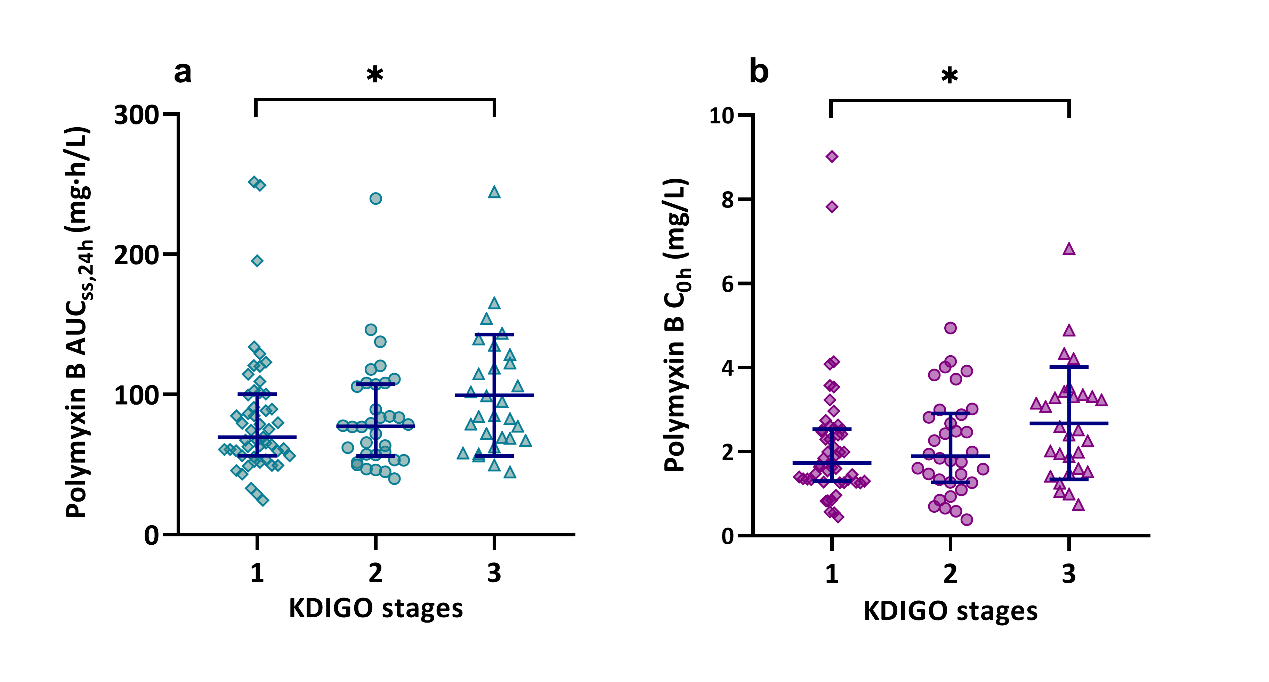


Figure S2. Scatterplot of polymyxin B AUC_ss,24h_ (a) and C_0h_ (b) stratified for different stages of acute kidney injury. AUC_ss,24h_, the area under the plasma concentration-time curve across 24 hours at steady state; C_0h_, trough concentration.


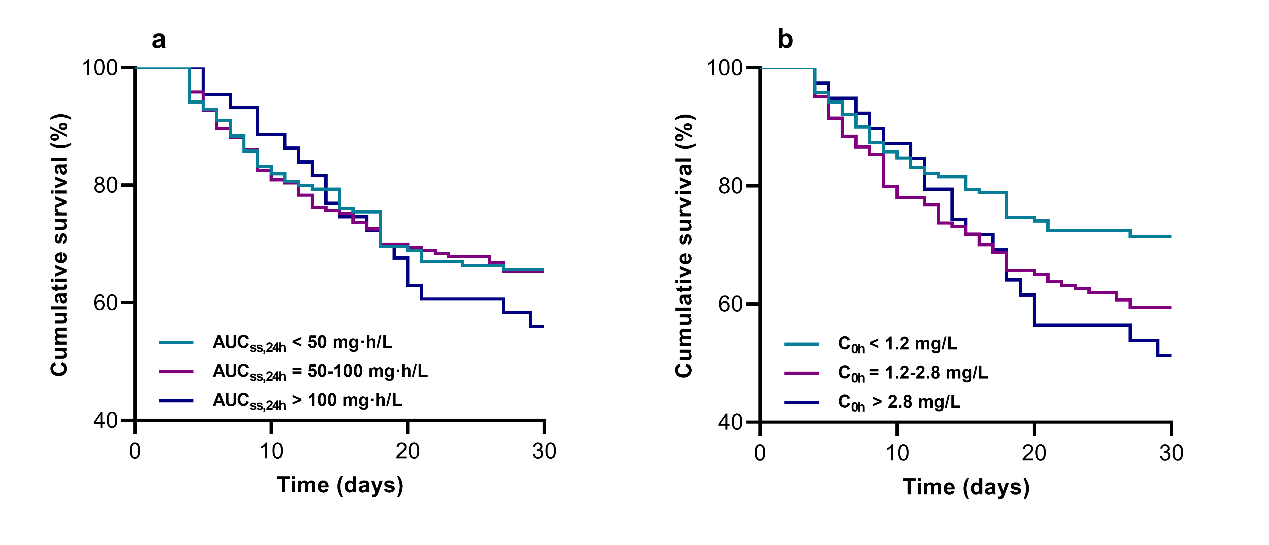


Figure S3. Kaplan-Meier estimates of survival to 30 days after administration of polymyxin B. Stratified by AUC_ss,24h_ (a) and C_0h_ (b). AUC_ss,24h_, the area under the plasma concentration-time curve across 24 hours at steady state; C_0h_, trough concentration.
